# Genome-Wide Identification of *TPL*/*TPR* Gene Family in Ten Cotton Species and Function Analysis of *GhTPL3* Involved in Salt Stress Response

**DOI:** 10.3390/genes16091072

**Published:** 2025-09-12

**Authors:** Ganggang Zhang, Jianguo Gao, Faren Zhu, Kailu Chen, Jiliang Fan, Lu Meng, Zihan Li, Shandang Shi, Hongbin Li

**Affiliations:** 1 Key Laboratory of Oasis Town and Mountain-Basin System Ecology of Bingtuan, College of Life Sciences, Shihezi University, Shihezi 832000, China; 19915233386@163.com (G.Z.); 15974804112@163.com (J.G.); ckl3224832883@163.com (K.C.); fanplanbeson@163.com (J.F.); mlulu@stu.shzu.edu.cn (L.M.); 2 Key Laboratory of Xinjiang Phytomedicine Resource and Utilization of Ministry of Education, College of Life Sciences, Shihezi University, Shihezi 832000, China; 3 Department of Civil, Environmental and Construction Engineering, College of Engineering and Computer Science, University of Central Florida, Orlando, FL 32816, USA; zihl2721@gmail.com

**Keywords:** cotton, *TPL*/*TPR* gene family, expression analysis, VIGS, salt stress tolerance

## Abstract

Background/Objectives: The TOPLESS (TPL) and TOPLESS-related (TPR) proteins represent a highly conserved class of transcriptional co-repressors in plants, playing pivotal roles in modulating growth, development, and stress responses through the repression of key transcriptional regulators. However, a comprehensive genome-wide analysis of the *TPL*/*TPR* gene family and its involvement in stress responses remains unexplored in cotton. Methods: In this study, 60 TPL/TPR genes were identified from the genomes of ten *Gossypium* species via bioinformatics approaches, and their protein physicochemical properties, gene structures, phylogenetic relationships, *cis*-regulatory elements, and expression profiles were characterized. Results: Chromosomal localization and collinearity analyses revealed that segmental duplication events have contributed to the expansion of the *TPL*/*TPR* gene family. Further examination of exon–intron architectures and conserved motifs highlighted strong evolutionary conservation within each *TPL*/*TPR* subgroup. Expression profiling demonstrated that *TPL*/*TPR* genes exhibit tissue-specific expression patterns, with particularly high transcript abundance in floral organs (e.g., petals and stigmas). *Cis*-element analysis suggested their potential involvement in multiple stress-responsive pathways. Notably, *GhTPL3* showed high constitutive expression across various tissues and under stress conditions, with the most pronounced up-regulation under salt stress. Functional validation via Virus-Induced Gene Silencing (VIGS) confirmed that *GhTPL3* silencing significantly impairs cotton salt stress tolerance, underscoring its critical role in abiotic stress adaptation. Conclusions: Our findings provide novel insights into the functional diversification and regulatory mechanisms of the *TPL*/*TPR* family in cotton, offering a valuable genetic resource for breeding stress-resilient cotton varieties.

## 1. Introduction

Cotton is a crucial economic crop for China’s textile industry. However, since 1993, global climatic and ecological changes have subjected cotton to severe abiotic stresses such as drought, high salinity, heat, and cold, significantly threatening cotton yield and fiber quality [1]. High-throughput cotton genome sequencing data have facilitated molecular breeding and evolutionary studies in cotton. In recent years, whole-genome sequence data for cotton species have been released, enabling systematic genome-wide identification and analysis of gene families [2].

*TOPLESS* (TPL) and *TOPLESS-related* proteins (TPR) are essential transcriptional co-repressors in plants, regulating diverse signaling pathways such as jasmonic acid, auxin, ethylene, brassinosteroid, and gibberellin signaling by recruiting chromatin-modifying complexes like histone deacetylases (HDACs) to repress target genes [3,4,5,6,7]. Structurally, *TPL*/*TPR* proteins contain a conserved *TOPLESS Domain* (*TPD*) for EAR motif binding, a *TPR* superhelical groove (formed by tandem TPR motifs within the CTLH and LisH domains) that recognizes EAR-containing repressors, and a LisH domain enabling dimerization to integrate signals from multiple transcriptional repressors. Some homologs further feature a WD40 repeat domain, broadening their interaction capacity with non-canonical transcription factors or auxiliary proteins [8,9,10,11]. Functionally, these domains collectively position *TPL*/*TPR* as central hubs for signal convergence, linking upstream repressive inputs to downstream epigenetic silencing mechanisms.

Studies indicate that this gene family forms a small multigene family in higher plant genomes, comprising 4 to 11 members. In *Arabidopsis thaliana*, the *TPL*/*TPR* family consists of five members: *TPR1*, *TPR2*, *TPR3*, *TPR4*, and *TPL* [12,13]. *TOPLESS* family members have recently been confirmed as key regulators in diverse gene repression mechanisms and play critical roles in auxin perception [8]. For instance, *TOPLESS-like* proteins (SlTPLs) identified in the tomato genome were used to construct a protein–protein interaction (PPI) network with *AUXIN*/*INDOLE-3-ACETIC ACID* (*Aux*/*IAA*) proteins via yeast two-hybrid assays. This PPI map revealed two distinct expression patterns: some *TOPLESS* isoforms interacted with most Aux/IAAs, while others exhibited only limited protein-binding capacity [14]. Research also demonstrates that the co-repressor *TOPLESS*, binding to the promoters of CUC3 and BRAVO via BES1, participates in brassinosteroid (BR)-mediated shoot organ boundary formation and root meristem cell division [15], and plays a key role in BZR1-regulated cell elongation [16]. Beyond development, *TPL*/*TPR* genes respond to biotic and abiotic stresses. For example, the cpr5 gene, encoding an *A. thaliana* nucleoporin mutant, activates autoimmunity partially mimicking Effector-Triggered Immunity (ETI). The NTR gene Exportin-4 (XPO4) functions as a genetic interactor of CPR5, which is a key regulator of plant immunity. The xpo4-cpr5 double mutant exhibits enhanced immune responses, resulting in seedling lethality. Under elevated salicylic acid (SA) conditions, the loss of XPO4 leads to the nuclear accumulation of *TPL*/*TPR* transcriptional corepressors. This accumulation contributes to SA-mediated defense amplification and enhances immune induction in the cpr5 mutant background [17].

In this study, *TPL*/*TPR* genes were systematically identified across ten cotton species, and their fundamental physicochemical properties, subcellular localization, phylogenetic relationships, chromosomal distribution, gene structures, conserved motifs, collinearity, and cis-acting elements in promoters were analyzed. We further examined the expression patterns of *TPL*/*TPR* genes across different tissues and under various abiotic stresses. Additionally, we investigated the response of the *Gossypium hirsutum* gene *GhTPL3* to salt stress using Virus-Induced Gene Silencing (VIGS). This study provides novel insights into the *GhTPL3* gene in cotton at the molecular level.

## 2. Results

### 2.1. Identification of TPL/TPR Genes and Analysis of Related Protein Physicochemical Properties

Using the conserved protein domain of *TPL*/*TPR* genes and the protein sequence of *A. thaliana* *TPL*/*TPR*, candidate sequences were screened from the ten cotton genomes (A1, A2, D1, D5, D10, AD1~5) employing the hidden Markov model search program (hmmsearch) and BLASTP [1]. The presence of the *TPL*/*TPR* domain in the candidate sequences was subsequently confirmed using Pfam and NCBI CDD [18]. After excluding sequences with incomplete *TPL*/*TPR* domains, a total of 60 *TPL*/*TPR* genes were identified, with counts of 4, 4, 4, 4, 4, 8, 8, 8, 8, and 8 genes originating from *Gossypium arboreum*, *Gossypium thurberi*, *Gossypium herbaceum*, *Gossypium turneri*, *Gossypium raimondii*,*Gossypium barbadense*, *G. hirsutum*,*Gossypium tomentosum*, *Gossypium darwinii*, and *Gossypium mustelinum,* respectively. Subsequently, these genes were named according to the sequential order of their chromosomal locations in the genomes of different corresponding species (Table S1). The amino acid lengths range from 1041 to 1336 aa; the lengths of the coding sequences (CDS) range from 3144 to 8336 bp; the protein molecular weight ranges from 115.99 to 148.06 kDa; and the pI value of the protein ranges from 6.22 to 7.35. In addition, the results of subcellular localization prediction show that 30 *TPL*/*TPR* genes are localized on the nucleus, 28 *TPL*/*TPR* genes are localized on the cytoplasm, while *GthTPL2* and *GrTPL3* are localized in the plasma membrane. The results indicate that in *G. hirsutum* there are significant differences in the amino acid length and relative molecular weight of *TPL*/*TPR* proteins, and they are generally weakly acidic, which is consistent with the general characteristics of *TPL*/*TPR* family proteins [19,20].

### 2.2. Analysis of TPL/TPR Gene Structure and Protein Conserved Motifs

**Figure 1:**
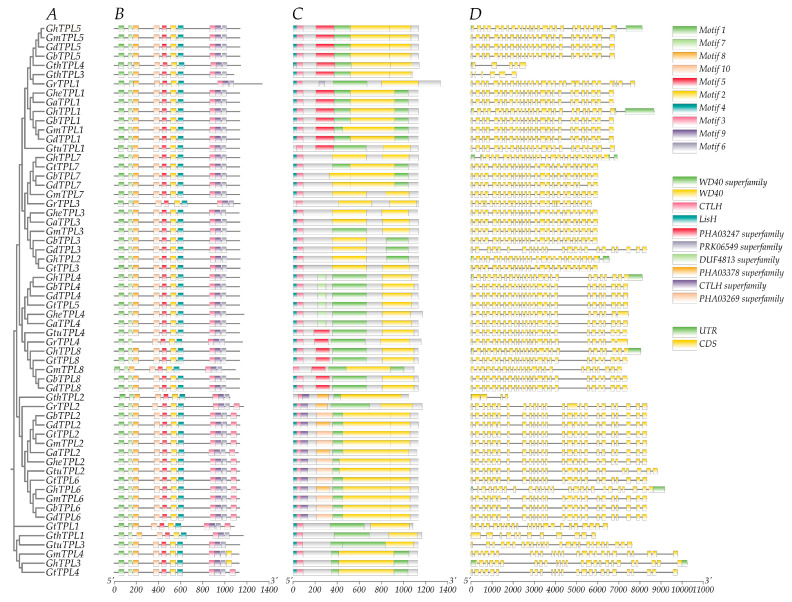
Sequence characteristics of *TPL*/*TPR* genes in ten cotton species. (**A**) Cotton *TPL*/*TPR* genes phylogenetic tree. (**B**) Identification of conserved protein motifs (1–10). (**C**) Characterization of conserved functional domains in *TPL*/*TPR* protein sequences. (**D**) Visualization map of *TPL*/*TPR* gene structure: exons (green), introns (black), and untranslated regions (yellow).

Protein sequences of the 60 identified *TPL*/*TPR* genes were aligned using ClustalX2, and a phylogenetic tree was constructed with MEGA11. Ten conserved motifs were identified via the MEME online program and visualized using TBtools V1.098. Results revealed that the 60 *TPL*/*TPR* genes clustered into six subgroups (Figure 1A). Each *TPL*/*TPR* protein contained 10 conserved motifs, with genes within the same branch exhibiting highly similar motif distribution patterns. Significant conservation was also observed across different subgroups. The motif arrangement is highly conserved, and the differences between subgroups are only minimal. (Figure 1B). Notably, Motif 1 containing the conserved N-terminal cysteine residue characteristic of *TPL*/*TPR* proteins was consistently present across all subgroups. All *TPL*/*TPR* proteins harbored the conserved *TPL*/*TPR* domain within their N-terminal regions (Figure 1C). These findings demonstrate that *TPL*/*TPR* genes within the same subgroup share similar motif architectures, while inter-subgroup domain variations are minimal. To further characterize gene structures, exon–intron organizations were determined using genome annotations from ten cotton species (Figure 1D). Analysis indicated that *TPL*/*TPR* genes generally possess numerous exons with clustered distributions. The majority contained either 25 exons (48/60, 80%) or 24 exons (4/60, 6.6%), displaying high structural conservation. The remaining genes exhibited variable exon counts: *G. thurberi* (diploid) genes *GthTPL4* and *GthTPL3* possessed the fewest exons (5 and 6, respectively), while *G. raimondii*’s *GrTPL1* contained the maximum (30 exons).

### 2.3. Phylogenetic Analysis of TPL/TPR Genes

**Figure 2:**
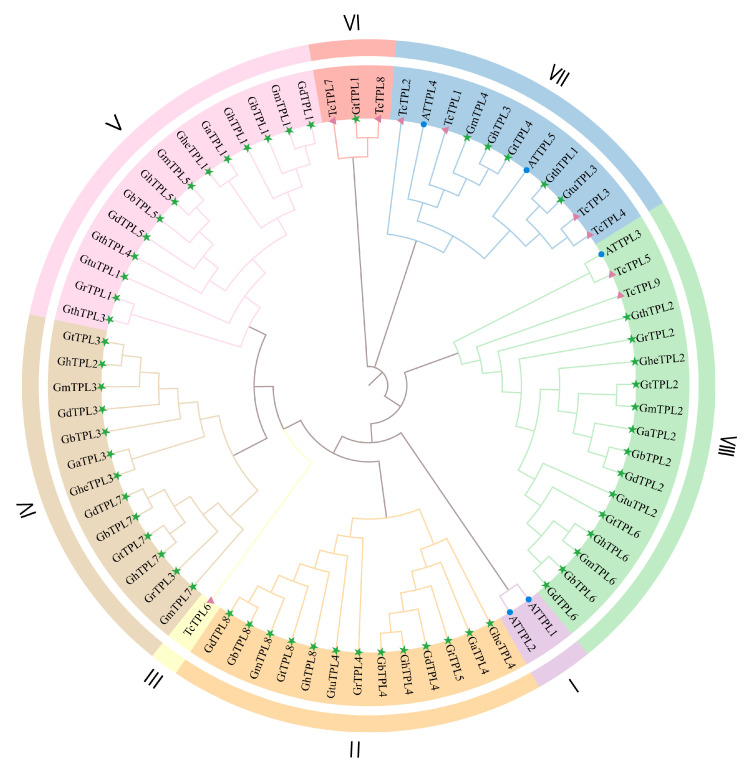
Phylogenetic tree of *TPL*/*TPR* gene family in three species. Green pentagrams identify members of the cotton *TPL*/*TPR* gene family, blue solid circles identify members of the *ATTPL* gene family, and purple triangles identify members of the *TcTPL* gene family.

A phylogenetic tree was constructed using MEGA11 based on protein sequences from 5 *A. thaliana TPL*/*TPR (ATTPL)*, 9 *Theobroma cacao TPL*/*TPR (TcTPL)*, and 60 cotton *TPL*/*TPR* genes (Figure 2). The analysis revealed that the *TPL*/*TPR* genes clustered into eight distinct subgroups (I–VIII), with subgroup VIII (17 members: 14 cotton, 2 *TcTPL*, and 1 *ATTPL*) and subgroup V (14 cotton genes) as the largest. Notably, only subgroups VII and VIII contained genes from all three species. Within these two subgroups, cotton *TPL*/*TPR* genes exhibited closer phylogenetic affinity and likely functional similarity to their *ATTPL* and *TcTPL* orthologs, while showing more distant relationships to members of other subgroups. Three phylogenetically isolated subgroups were identified: subgroup I exclusively contained two *ATTPL* genes, subgroup III comprised a single *TcTPL* gene, and subgroup VI included two *TcTPL* genes and one cotton gene. This distribution pattern indicates that *ATTPL1*, *ATTPL2*, and *TcTPL6* are evolutionarily conserved with distinct trajectories compared to most cotton *TPL*/*TPR* homologs. Collectively, these results demonstrate differential evolutionary patterns and varying levels of sequence conservation among *TPL*/*TPR* genes across plant species [21,22].

### 2.4. Chromosomal Localization and Interspecies Collinearity Analysis of TPL/TPR Genes

**Figure 3:**
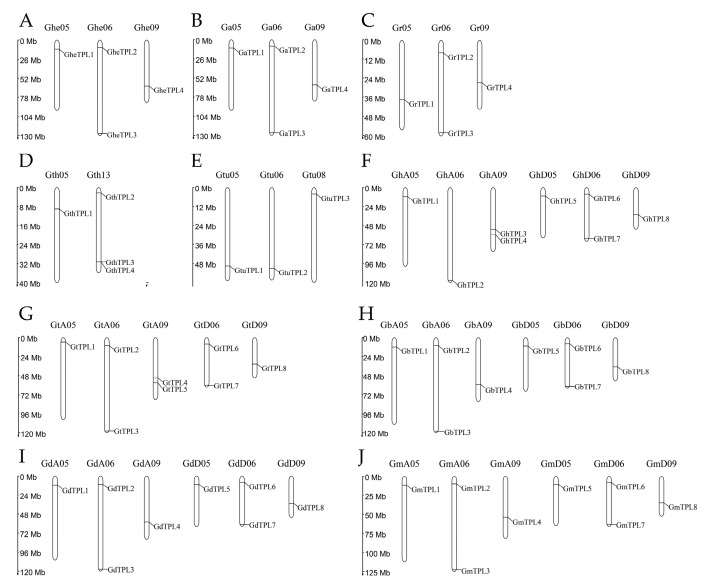
Chromosomal mapping of the TPL/TPR genes. (**A**) *G. herbaceum*. (**B**) *G. arboreum*. (**C**) *G. raimondii*. (**D**) *G. thurberi*. (**E**) *G. turneri*. (**F**) *G. hirsutum*. (**G**) *G. barbadense*. (**H**) *G. tomentosum*. (**I**) *G. mustelinum*. (**J**) *G. darwinii*.

**Figure 4:**
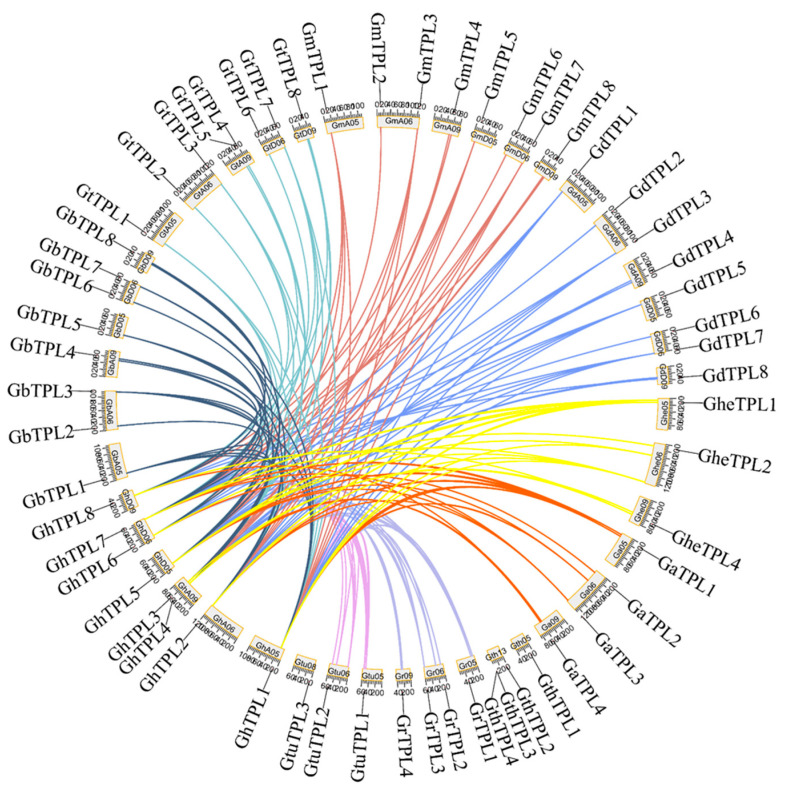
Collinearity between *G. hirsutum* and nine cotton species based on homologous gene pair analysis. Connecting lines of the same color represent homologous gene pairs between two species.

Gene family expansion mainly occurs through three mechanisms: tandem duplication, chromosomal segmental duplication/whole-genome recombination, and retrotransposition [23,24]. To understand the expansion of the *TPL*/*TPR* gene family in different cotton varieties, chromosomal localization (Figure 3) and collinearity analysis (Figure 4) of the 60 *TPL*/*TPR* genes were conducted in this study. *TPL*/*TPR* genes are distributed across 43 chromosomes, with a relatively uniform distribution. Most chromosomes contain only one *TPL*/*TPR* gene, while chromosomes GheA06, GaA06, GhA09, GdA06, GbA06, GtA06, GmA06, GrD06, GhD06, GtD06, GbD06, and GmD06 each have two *TPL*/*TPR* genes; only chromosome *GtD13* has three *TPL*/*TPR* genes. When two or more genes are located on the same chromosome, tandem duplication events occur. Tandem duplication events were observed but were less frequent than segmental duplications; only chromosomes GtA09 and GhA09 each have a tandem event with two genes, and chromosome GthD13 has a tandem event with three genes. In addition, segmental duplication or Whole-Genome Duplication (WGD) events occur between chromosomes.

To clarify the driving force behind the expansion of the *TPL*/*TPR* gene family, the duplication events of *TPL*/*TPR* genes in 10 cotton varieties were analyzed. The *TPL*/*TPR* gene pairs obtained through collinearity analysis were visualized using a circular graph (Figure 4). The analysis of collinearity between tetraploid and diploid cotton was also conducted. It was found that a total of 183 gene pairs were generated among the *TPL*/*TPR* genes of the ten cotton varieties. Based on this, it is inferred that the evolution and amplification of the *TPL*/*TPR* gene family in cotton are primarily driven by segmental duplication events, which further supports segmental duplication as the main driving factor for *TPL*/*TPR* gene amplification in cotton.

### 2.5. Cis-Regulatory Element Analysis of the TPL/TPR Gene Family

All promoters contained three conserved eukaryotic regulatory motifs CAAT-box, Box 4, and MYC, which modulate transcriptional activity through transcription factor binding. Meristem-responsive elements were absent in only five genes, suggesting roles in cotton development and reproduction. Gibberellin-responsive elements (GAREs) occurred as single copies in 12 genes, indicating potential functions in stress adaptation. Most promoters featured multiple hormone-responsive elements, with greater than 30 distinct motifs present in the majority of the promoters. *GbTPL2* showed minimal diversity (12 types) while *GmTPL5* exhibited maximal complexity (22 types) (Figure S1A). Additionally, while the primary elements associated with growth and development are well-established, a considerable number of stress-responsive elements have also been identified, including low-temperature responsive elements, drought induction motifs, and salt-responsive elements. Collectively, these findings indicate that *TPL*/*TPR* genes likely play significant roles in cotton growth, reproductive development, and stress tolerance (Figure S1B).

### 2.6. Expression Profiles of TPL/TPR Genes in Different Tissues and Under Abiotic Stresses

**Figure 5:**
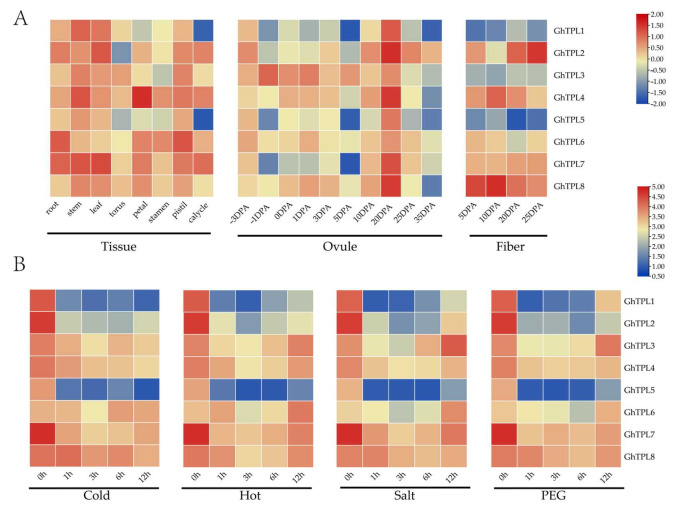
Expression pattern diagram of *GhTPL1–8* in different tissues and under different abiotic stress treatments of *G*. *hirsutum*. (**A**) Analysis of *TPL*/*TPR* tissue expression patterns. (**B**) Analysis of *TPL*/*TPR* stress expression patterns.

To clarify the biological functions of *GhTPL* genes, this study characterized the spatio-temporal regulatory patterns of eight *GhTPL* members in different tissues and under various abiotic stresses. Through systematic screening, key functional genes were identified. A clustered heatmap depicting the tissue-specific expression profiles was generated using TBtools V1.098 to analyze the expression patterns of *TPL*/*TPR* genes across various tissues and under abiotic stresses (Figure 5). *GhTPL1*~*GhTPL8* exhibited high expression in both vegetative and reproductive organs, suggesting multifunctional roles. During root development (24–120 h), all *GhTPL1-GhTPL8* genes peaked at 96 h. *GhTPL4* showed the highest expression in roots and petals, followed by *GhTPL7*. During ovule development, *GhTPL* expression peaked at 20 days post-anthesis (DPA). Notably, *GhTPL2*, *GhTPL4*, *GhTPL7*, and *GhTPL8* displayed elevated expression during fiber development (20–25 DPA). These four homologous genes exhibited similar temporal expression patterns, suggesting that they may have synergistic roles in cotton fiber development (Figure 5A). Under abiotic stress treatments, *GhTPL3*~*4* and *GhTPL6*~*8* showed induced expression, predominantly peaking at 12 h post-treatment. *GhTPL3* and *GhTPL8* maintained consistently high expression across multiple timepoints, particularly at 12 h, whereas *GhTPL1* and *GhTPL5* demonstrated relatively weak stress responsiveness (Figure 5B).

### 2.7. GhTPL3-Silenced Cotton Plants Showed High Sensitivity to Salt Stress

**Figure 6:**
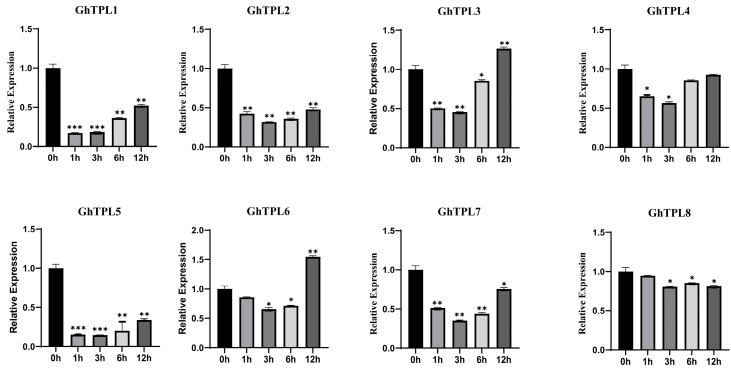
RT-qPCR verification of the expression levels of *GhTPL1*~*8* in *G. hirsutum* variety TM-1 under salt stress treatment. The standard deviation (SD) of three biological replicates is represented by error bars, with statistical significance indicated by * (*p* < 0.05), ** (*p* < 0.01), and *** (*p* < 0.001).

**Figure 7:**
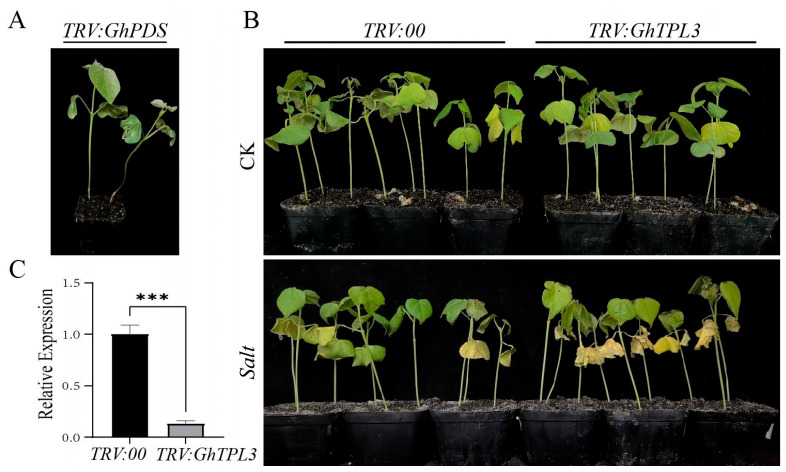
Functional validation of *GhTPL3* silencing in cotton under salt stress. (**A**) Phenotype of positive control *TRV*:*GhPDS* plants showing characteristic photobleaching symptoms. (**B**) Phenotypic comparison of *GhTPL3-silenced G. hirsutum* plants before and after 350 mM NaCl treatment. (**C**) RT-qPCR analysis of *GhTPL3* expression levels in leaves of VIGS-silenced plants. *** (*p* < 0.001) denotes significant differences.

The expression levels of *GhTPL1*~*8* in *G. hirsutum* variety TM-1 under salt stress were analyzed by RT-qPCR. The expression levels of *GhTPL1*~*7* genes all showed a pattern of first decreasing and then increasing, while only *GhTPL8* did not respond positively to salt stress (Figure 6). Interestingly, the expression level of *GhTPL3* under salt stress was much higher than that of the other seven genes. Therefore, *GhTPL3* was selected as a candidate gene for further functional studies in this study. The required primers were designed (Table S2). To investigate the function of *GhTPL3,* we employed the VIGS method to silence this gene in G. hirsutum variety TM-1, and verified the silencing efficiency in leaves via RT-qPCR. In the VIGS experiment, *TRV*:*GhPDS* was used as the positive control, and *TRV*:*00* as the negative control. The lower epidermis of cotton leaves was infected. Several weeks after *Agrobacterium* infection, the albino phenotype was visible on the cotton leaves infiltrated with the positive control *TRV*:*PDS* (Figure 7A), indicating that the VIGS procedure was correct and effective. Then, RT-qPCR was performed to detect the expression level of *GhTPL3* gene. Compared with the control *TRV*:*00*, the expression level of *GhTPL3* gene was much lower, indicating the success of gene silencing (Figure 7C). The experimental group was treated with high-concentration salt. As time went on, the leaves gradually wilted, drooped due to water loss, and the leaf edges curled. After 72 h of salt stress, the two cotyledons of *TRV*:*GhTPL3*-silenced plants turned yellow, and even became scorched in severe cases (Figure 7B). The experiment showed that *GhTPL3* is an important positive regulator under salt stress.

## 3. Discussion

A gene family is defined by a set of genes originating from a common ancestor, with multiple copies of the gene produced through gene duplication events [25,26,27]. Transcriptional repression serves as a critical element within a plant’s genetic toolkit, playing a pivotal role in spatial and temporal gene expression, responses to environmental stimuli, homeostasis, and other biological processes [28]. The *TPL*/*TPR* gene family is a specific type of transcriptional repressor in plants. To date, *TPL*/*TPR* genes have been identified in numerous plant species including oilseed rape [29], rice [30], maize [31], chrysanthemum [32], and tomato [14]. Cotton is a significant natural fiber crop, with limited research on the *TPL*/*TPR* gene family in this species. This study conducted a genome-wide analysis of *TPL*/*TPR* genes across ten cotton species, identifying a total of 60 *TPL*/*TPR* genes. (Table S1). In general, the lower the ploidy level of a plant, the fewer the number of genes in its *TPL*/*TPR* gene family [33,34,35]. For example, among monocotyledonous species, two *TPL*/*TPR* homologous proteins *(REL1*~*2)* have been identified in diploid maize so far, and three (*OsTPP1*~*3*) in diploid rice. Among dicotyledonous species, there are 6 *TPL*/*TPR* genes in diploid tomato (*SLTPL1*~*6*) and 18 in tetraploid rapeseed (*BraA1*~*A9, BnaA1*~*A9*) [34]. In this study, the number of *TPL*/*TPR* genes in tetraploid cotton is twice that in diploid cotton, which is consistent with the known evolutionary relationship of cotton. This may be due to the genome-wide expansion of the ten cotton species during evolution, leading to the amplification of *TPL*/*TPR* genes in tetraploid cotton. The exon–intron structures of *TPL*/*TPR* genes in the ten cotton species are basically the same. Most *TPL*/*TPR* proteins (30/60, 50%) are subcellularly localized in the nucleus (Table S1), which is consistent with the regulatory role of transcription factors in the nucleus. The *TPL*/*TPR* proteins from the ten cotton species were all unstable, hydrophilic, and weakly acidic proteins (Table S1), with no significant differences. The phylogenetic tree of the 60 *TPL*/*TPR* proteins was divided into eight distinct clusters (Figure 2). The members in subclusters II, IV, and V only included cotton *TPL*/*TPR* proteins; subcluster III contained only one member, *T. cacao TcTPL6*; and subcluster I contained only two members, *A. thaliana ATTPL1–2*. In contrast, subclusters VII and VIII included members from three species: *A. thaliana*, *T. cacao,* and cotton, suggesting that the rapid expansion of *TPL/TPR* genes appears to have occurred following the divergence of monocotyledonous and dicotyledonous plants. In cluster VI, the *GtTPL1* gene showed high homology with *TcTPL7* and *TcTPL8*, indicating that they may have similar functions.

*Cis*-acting elements play a pivotal role in the regulation of gene expression and transcriptional processes [24]. In this study, it was found that the regulatory regions of cotton *TPL*/*TPR* genes contain numerous *cis*-elements responsive to stresses and hormones, indicating their potential importance in mediating cotton’s response to abiotic stresses and in hormone regulatory pathways (Figure S1). In *A. thaliana*, the high-temperature responsive element DEAR4 confers heat tolerance to plants by recruiting TOPLESS in the nucleus of *A. thaliana* [33]. In poplar, *TPL*/*TPR* is recruited by transcription factors such as HSF, which affects the expression of jasmonic acid synthase genes (*LOX*, *AOS*) and regulates salt tolerance [16,33]. Subsequently, through the expression profile heatmap (Figure 5) and RT-PCR analysis of *GhTPL* genes under different abiotic stress treatments (Figure 6), it was found that *GhTPL3* and *GhTPL6* were induced by salt, high-temperature, and drought stresses, showing a significantly up-regulated expression pattern. In particular, the expression level of *GhTPL3* under salt stress was much higher than that of the other *GhTPL* genes. According to previous studies, the *GhTPL3* gene is localized in the nucleus, and its promoter region contains various hormone-responsive elements and abiotic stress-responsive cis-acting elements, such as the CGTCA-motif (involved in methyl jasmonate response), TGA-element (salicylic acid response), ABRE (abscisic acid response), GA-motif (gibberellin response), as well as MYB binding sites and TC-rich repeats (related to defense and stress responses). Therefore, *GhTPL3* was selected as a candidate gene for subsequent functional studies. We silenced the *GhTPL3* gene using VIGS and then treated the plants under salt stress. RT-PCR verification showed that the expression level of *GhTPL3* was significantly reduced, indicating successful silencing (Figure 7A). Following salt treatment, the *GhTPL3*-silenced plants exhibited more pronounced leaf wilting, desiccation, and curling compared to the control plants (Figure 7B). Preliminary evidence indicates that *GhTPL3*, a key salt stress-responsive gene in cotton, possesses a promoter enriched with various hormone and stress-related *cis*-elements. This suggests its potential to orchestrate salt stress signaling via a putative complex regulatory network.

This study conducted a comprehensive examination of the evolutionary and functional attributes of the *TPL*/*TPR* gene family in cotton, and *GhTPL3* was identified as a key regulatory factor induced by salt stress. Future work can further verify the function of *GhTPL3* and deepen the understanding of its regulatory mechanisms, as well as explore the functional redundancy and synergistic mechanisms of other subfamily members, by constructing transgenic lines using protein interaction technology and transgenic technology, combined with hormone signaling pathways. These findings not only provide insights into the functional differences and synergistic mechanisms among *TPL*/*TPR* subfamilies in cotton, but also offer new research directions for breeding high-quality, stress-tolerant cotton varieties.

## 4. Materials and Methods

### 4.1. Plant Materials and Experimental Treatments

RT-qPCR experimental treatment: *G. hirsutum* (TM-1) was planted in a culture chamber with a 16 h/8 h (light/dark) cycle, temperature controlled at 25 °C, and relative humidity maintained at 60–70%. After the cotton seedlings emerged from the soil, the seedling covers were removed. After 15 days of continued cultivation, vegetative-stage plants with consistent growth were subjected to the same salt stress treatment (350 mM NaCl). Leaf samples were collected at various time points post-treatment (0, 1, 3, 6, and 12 h after stress induction). The samples were immediately frozen and stored at −80 °C to maintain RNA integrity until nucleic acid extraction. Primers required for RT-qPCR were designed (primers in Supplementary Table S2). Each experimental group consisted of 3–5 biological replicates (different plants), each measured with three technical replicates. Data analysis and visualization were conducted using GraphPad Prism (version 10.1.1).

VIGS experimental treatment: The extracted RNA was reverse-transcribed into cDNA. Specific primers for *GhTPL3* were designed (primers in Supplementary Table S3). Using cDNA as the template, PCR amplification was performed with *GhTPL3-VIGS-F* and *GhTPL3-VIGS-R* as the upstream and downstream primers, respectively. The products were recovered and purified by agarose gel electrophoresis. The PCR product was ligated into the tobacco rattle virus vector (*TRV*:*00*) empty vector using *EcoR* I and *Kpn* I restriction enzyme sites. The *TRV*:*GhTPL3* construct was introduced into *Agrobacterium tumefaciens* (*A. tumefaciens*) *GV3101* competent cells via the freeze–thaw method. When *G. hirsutum* TM-1 seedlings reached the third true leaf stage, the resuspensions of the negative control (*TRV*:*00*), positive control (*TRV*:*GhPDS*), and experimental group (*TRV*:*GhTPL3*) were mixed at a 1:1 ratio for later use. The back of the cotyledons was gently pricked with a 1 mL sterile syringe needle, and the head of a needleless syringe was aligned with the wound on the cotyledons to fill the cotyledons with the bacterial solution. After infection, the cotton plants were cultured in the dark overnight, and then transferred to a culture chamber at 25 °C under normal light conditions for 10–15 days of cultivation. When the leaves of the positive control (*TRV*:*PDS*) plants showed albinism (Figure 7A), leaves from the experimental group *TRV*:*GhTPL3* were collected to verify the silencing efficiency (Figure 7C). Subsequently, the experimental group was subjected to 72 h salt stress treatment (350 mM NaCl) and phenotypic differences between the experimental group (*TRV*:*GhTPL3)* and negative control (*TRV*:*00*) were observed (Figure 7B). To ensure the accuracy and reliability of the experimental data, three biological replicates were established for each experimental material, with each replicate containing three seedlings.

### 4.2. The Identification Process Construction of Cotton TPL/TPR Genes and Subcellular Localization

Genomic DNA sequences, protein sequences, and annotation files for cotton were retrieved from the CottonMD database. Corresponding data for *T. cacao* and *A. thaliana* were obtained from the Ensembl Plants database and TAIR database. The hidden Markov model (HMM) profile for the conserved *TPL*/*TPR* domain was downloaded from the Pfam database. Local HMM searches were performed using hmmsearch with a stringent E-value cutoff of 1 × 10^−1^, identifying 60 candidate protein sequences containing the *TPL*/*TPR* domain. To minimize potential errors arising from mis-prediction, BLASTP searches were conducted against ten cotton protein databases using five known *A. thaliana TPL*/*TPR* protein sequences as queries. Candidate sequences were further validated for the presence of the conserved *TPL*/*TPR* domain using both Pfam and the NCBI CDD. This comprehensive approach ultimately identified 60 bona fide *TPL*/*TPR* genes in cotton [2,18].

The molecular weight (MW), amino acid length (aa), and isoelectric point (pI) of the 60 *TPL*/*TPR* proteins were predicted using the ExPASy Compute pI/Mw tool. Subcellular localization predictions were performed by submitting the protein sequences to WoLF PSORT, and predictions were selected based on the highest confidence scores provided by the database.

### 4.3. Multiple Species TPL/TPR Protein Sequence Alignment and Phylogenetic Tree Analysis

Protein sequences of *TPL*/*TPR* genes from cotton, *A. thaliana*, and *T. cacao* were aligned using ClustalX (version 2.1). The resulting multiple sequence alignment was imported into MEGA11 software. A maximum likelihood (ML) phylogenetic tree was constructed using the aforementioned multiple sequence alignment [31]. The bootstrap method with 1000 replicates and an 80% partial deletion cutoff for site coverage was used to assess branch support. The final phylogenetic tree was visualized and annotated using TBtools.

### 4.4. Construction of Cotton TPL/TPR Gene Chromosome Map and Intraspecific Collinearity Visualization Analysis

Chromosome lengths were determined using SeqKit. The physical positions of *TPL*/*TPR* genes were extracted from cotton genome annotation files and mapped to chromosomes using MG2C.

Intraspecies synteny analysis of *TPL*/*TPR* genes across the cotton genomes was performed using MCScanX-transposed, utilizing nucleotide sequence files and genome annotation files as input. The synteny results were visualized using TBtools.

### 4.5. Cotton TPL/TPR Gene Promoter Sequence Extraction and PlantCARE Cis-Element Prediction Analysis

Promoter regions, defined as the 2000 bp upstream sequences of the transcription start site for each *TPL*/*TPR* gene in ten cotton species, were extracted using SeqKit. The promoter sequences were submitted to the PlantCARE database to predict *cis*-acting regulatory elements [23]. The identified elements were categorized, their frequencies were statistically analyzed, and the distribution patterns were visualized using TBtools.

### 4.6. TPL/TPR Expression Pattern Analysis

Expression data of the published *G. hirsutum TPL*/*TPR* genes were obtained from the Cotton MD website. These data sets covered expression profiles across different tissues and under different abiotic stresses. Microsoft Excel was used to process the expression-level data (FPKM: Fragments Per Kilobase of transcript per Million mapped reads). For experimental validation, RT-qPCR was performed with *GhUBQ7* as the internal control gene, and the relative expression levels were quantified using the 2^−ΔΔCt^ method. The primer sequences are provided in Supplementary Table S2. Finally, a clustered heatmap was generated using TBtools.

## 5. Conclusions

This study systematically identified and characterized the *TPL*/*TPR* gene family across multiple cotton species through comprehensive bioinformatics and comparative genomics analyses. Integrated expression profiling revealed *GhTPL3* as a crucial regulator of cotton’s salt stress response. Functional validation via VIGS demonstrated that *GhTPL3* knockdown substantially compromises cotton’s salt tolerance. These breakthroughs deepen mechanistic insights into *TPL*/*TPR* genes functions in cotton, simultaneously providing pivotal targets for molecular breeding and foundational biological research.

## Data Availability

Data is contained within this article or the Supplementary Materials.

## Supplementary Materials

The following supporting information can be downloaded at https://www.mdpi.com/article/10.3390/genes16091072/s1: Table S1. Physicochemical properties of *TPL*/*TPR* gene family members. Table S2. Primer sequences of qRT-PCR. Table S3. VIGS-related primer sequences. Table S4. The database and online website addresses appearing in this article. Figure S1. Characterization of cis-acting elements of *TPL*/*TPR* gene family.
