# Supplementary material for: Genome-Wide Identification of TPL/TPR Gene Family in Ten Cotton Species and Function Analysis of GhTPL3 Involved in Salt Stress Response
Source: Genes (Basel). 2025 Sep 12;16(9):1072. doi: 10.3390/genes16091072 (PMC12469280; doi:10.3390/genes16091072)
Supplement: Supplementary file 1 [file genes-16-01072-s001.zip › genes-3844765-supplementary Figure S1.pdf]

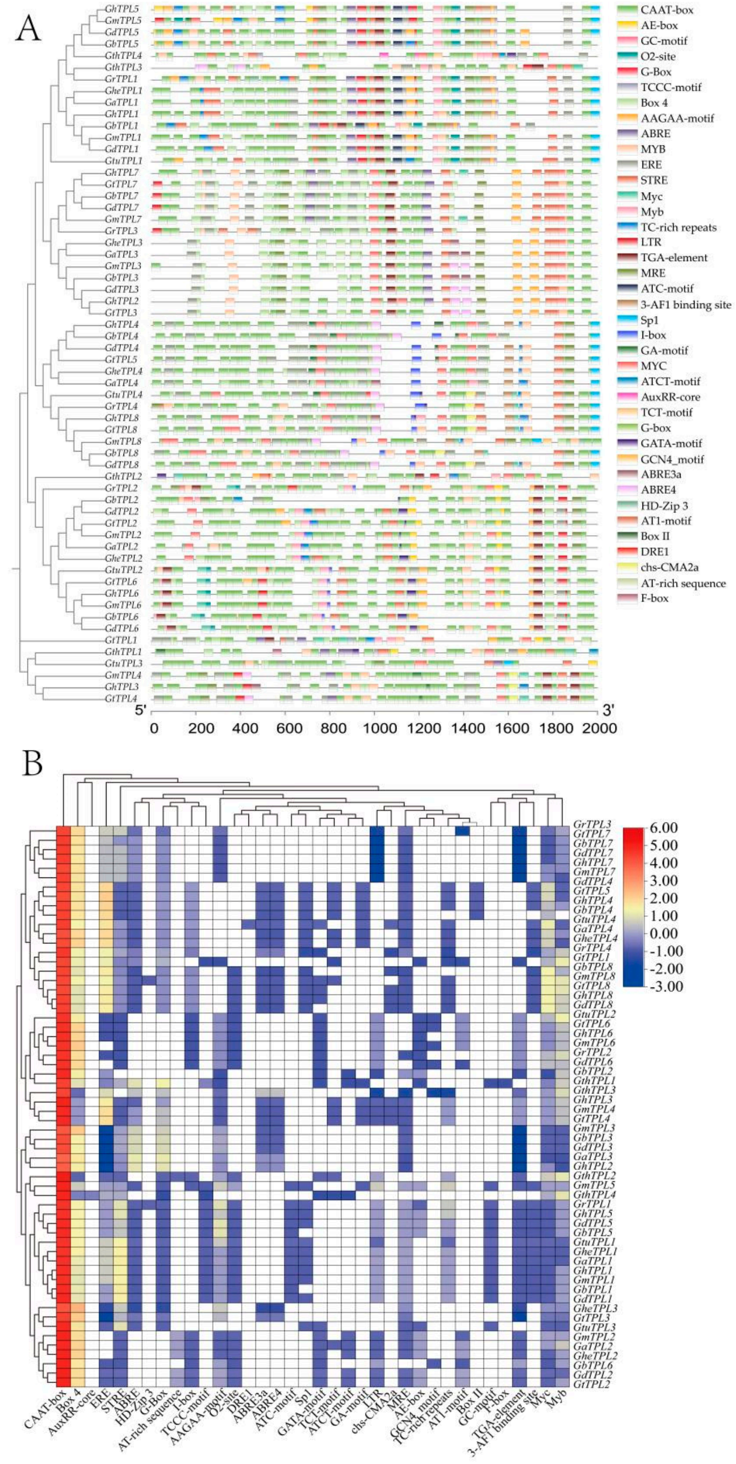

Figure S1 Characterization of cis-acting elements of *TPL*/*TPR* gene family. (A) Cis-acting element analysis of *TPL*/*TPR* gene family promoter in cotton. (B) Cis-acting element heatmap of *TPL*/*TPR* gene family promoter in cotton. Rows represent genes, columns represent different types of response elements, and the color gradient indicates the number of elements (red for high abundance and blue for low abundance).
